# Rotenone Induces Parkinsonism with Constipation Symptoms in Mice by Disrupting the Gut Microecosystem, Inhibiting the PI3K-AKT Signaling Pathway and Gastrointestinal Motility

**DOI:** 10.3390/ijms26052079

**Published:** 2025-02-27

**Authors:** Li Liu, Yan Zhao, Weixing Yang, Yuqin Fan, Lixiang Han, Jun Sheng, Yang Tian, Xiaoyu Gao

**Affiliations:** 1 Yunnan Key Laboratory of Precision Nutrition and Personalized Food Manufacturing, Yunnan Agricultural University, Kunming 650201, China; 2022210085@stu.ynau.edu.cn (L.L.); shengj@ynau.edu.cn (J.S.); tianyang@ynau.edu.cn (Y.T.); 2 College of Food Science and Technology, Yunnan Agricultural University, Kunming 650201, China; 2021110006@stu.ynau.edu.cn (W.Y.); 15348746213@163.com (Y.F.); 15368025745@163.com (L.H.); 3 Engineering Research Center of Development and Utilization of Food and Drug Homologous Resources, Ministry of Education, Yunnan Agricultural University, Kunming 650201, China; 4 Division of Science and Technology, Yunnan Agricultural University, Kunming 650201, China; 2021013@ynau.edu.cn

**Keywords:** movement disorders, non-motor symptoms, inflammation, barrier, constipation, gut microbiota, *Akkermansia*, *Staphylococcus*, *Acinetobacter*, *Lactobacillus*, *Bifidobacterium*, *Solibacillus*, *Eubacterium xylanophilum*-group

## Abstract

Parkinson’s disease (PD) is one of the most common neurodegenerative diseases. Constipation is a prodromal symptom of PD. It is important to investigate the pathogenesis of constipation symptoms in PD. Rotenone has been successfully used to establish PD animal models. However, the specific mechanism of rotenone-induced constipation symptoms is not well understood. In this work, we found that constipation symptoms appeared earlier than motor impairment in mice gavaged with a low dose of rotenone (30 mg/kg·BW). Rotenone not only caused loss of dopaminergic neurons and accumulation of α-synuclein, but also significantly reduced serum 5-HT levels and *5-HTR4* in the striatum and colon. The mRNA expression of aquaporins, gastrointestinal motility factors (*c-Kit*, *Cx43*, *smMLCK* and *MLC-3*) in mouse colon was also significantly regulated by rotenone. In addition, both colon and brain showed rotenone-induced inflammation and barrier dysfunction; the PI3K/AKT pathway in the substantia nigra and colon was also significantly inhibited by rotenone. Importantly, the structure, composition and function of the gut microbiota were also significantly altered by rotenone. Some specific taxa were closely associated with motor and constipation symptoms, inflammation, and gut and brain barrier status in PD mice. *Akkermansia*, *Staphylococcus* and *Lachnospiraceae_UCG*—*006* may play a role in exacerbating constipation symptoms, whereas *Acinetobacter*, *Lactobacillus*, *Bifidobacterium*, *Solibacillus* and *Eubacterium_xylanophilum*_groups may be beneficial in stimulating gastrointestinal peristalsis, maintaining motor function and alleviating inflammation and barrier damage in mice. In conclusion, low-dose rotenone can cause parkinsonism with constipation symptoms in mice by disrupting the intestinal microecosystem and inhibiting the PI3K-AKT pathway and gastrointestinal motility.

## 1. Introduction

Parkinson’s disease (PD) is one of the most prevalent neurodegenerative diseases, with an escalating incidence on an annual basis. The pathology of PD is characterized by a progressive degeneration of dopaminergic (DA) neurons in the substantia nigra compacta (SNpc) and misfolded aggregates of α-synuclein (α-Syn) within the remaining DA neurons to form Lewy bodies, which subsequently leads to a reduction in striatal dopamine [1,2]. Typical motor symptoms include slow movements, rigidity, resting tremor, and postural instability in patients with PD. In addition, PD patients clinically present with a variety of non-motor symptoms (NMS), including gastrointestinal (GI) dysfunction, cognitive deficits, sleep disturbances, and affective disorders.

Constipation is one of the most common NMS in PD patients. The incidence of constipation is as high as 24.6–63.0% in PD patients, which seriously affects the quality of life of patients [3]. Numerous studies have reported that GI dysfunction often precedes motor symptoms by many years [4]. Recent studies have suggested that α-Syn may first accumulate in the enteric nervous system (ENS) and then spread to the brainstem, leading to neurodegeneration and movement disorders. This finding helps explain why PD GI symptoms often precede PD dyskinesia [5]. However, the “intestinal origin” hypothesis is currently debated regarding the pathophysiology of PD. Consequently, research into the pathogenesis of constipation symptoms in PD is imperative for the development of effective treatment strategies.

A stable gut microbial composition plays an important role in maintaining individual health. The close connection between the brain and the GI system is influenced by the gut microbiota, which can positively regulate brain development and behavior through the gut–brain axis [6]. It has been posited that PD patients exhibit a diminished population of beneficial bacteria, such as *Lactobacillus* and *Bifidobacteria*, accompanied by an augmented presence of potentially deleterious bacteria, including *Clostridium* and *Enterococcus*, within their intestinal microbiota. This imbalance in the composition of the microbiota has been hypothesized to initiate a series of pathological processes, such as GI dysfunction and inflammatory responses [7]. In contrast, healthy mice receiving intestinal microbiota transplants from PD donors exhibit motor dysfunction [8]. Constipation is frequently accompanied by dysbiosis of the intestinal microbiota, which is typified by a decrease in the relative abundance of beneficial microbes (e.g., *Bifidobacterium* and *Lactobacillus*) and a relative increase in pathogenic microbes [9]. Consequently, intestinal microbiota can be regarded as a co-pathologic mechanism for PD and constipation.

Recent studies have indicated that the PI3K-AKT signaling pathway may also be a common target for the treatment of both PD and constipation. For example, hydrogen-saturated saline has been shown to exert neuroprotective effects by activating PI3K/AKT/mTOR pathway-mediated autophagy in the early and middle stages of rotenone-induced PD in rats [10]. Pterostilbene alleviates constipation in slow constipated rats by reducing Interstitial Cell of Cajal (ICC) apoptosis through activation of the PI3K-AKT signaling pathway [11]; Electroacupuncture also promotes autophagy in enteric neuroglia through activation of the PI3K/AKT/mTOR signaling pathway, thereby alleviating functional constipation in mice [12].

Gut microbiota and its metabolites may also affect the health of the host through the PI3K-AKT pathway. One study found that gut microbiota and butyrate were involved in the effects of a high-salt diet on synapses and memory, and that the PI3K-AKT pathway may mediate memory impairment in mice on a high-salt diet [13]. Short-chain fatty acids and indole derivatives can modulate intestinal immunity and restore the intestinal barrier by activating the PI3K-AKT pathway [14]. Furthermore, intestinal microbiota may indirectly affect NO production by influencing the PI3K-AKT pathway [15]. Excess NO can directly relax intestinal smooth muscle, slowing GI peristalsis and prolonging colonic transit, leading to constipation [16]. However, to date, few studies have focused on the roles of both intestinal microbiota and the PI3K/AKT pathway in the generation of constipation symptoms in PD.

Rotenone is a neurotoxin that is widely used because it induces a mouse model of PD with pathologic changes and symptomatic manifestations similar to those of human PD [17]. Rotenone has been demonstrated to induce abnormal activation and aggregation of α-syn in the intestinal tract, thereby causing damage to the ENS and affecting the normal function of the intestinal tract [18]. However, the specific role of the gut microenvironment and the PI3K-AKT signaling pathway in rotenone-induced GI dysmotility and constipation symptoms in PD remains unclear. For this purpose, this study successfully established a mouse model of PD with constipation under the induction of low-dose rotenone, and, on this basis, explored the potential mechanism of PD with constipation symptoms from the perspectives of GI motility factors, the PI3K-AKT signaling pathway, intestinal inflammation and barriers, and intestinal microbiota.

## 2. Results

### 2.1. Constipation Symptoms Induced by Rotenone Precede Dyskinesia in Mice 

**Figure 1:**
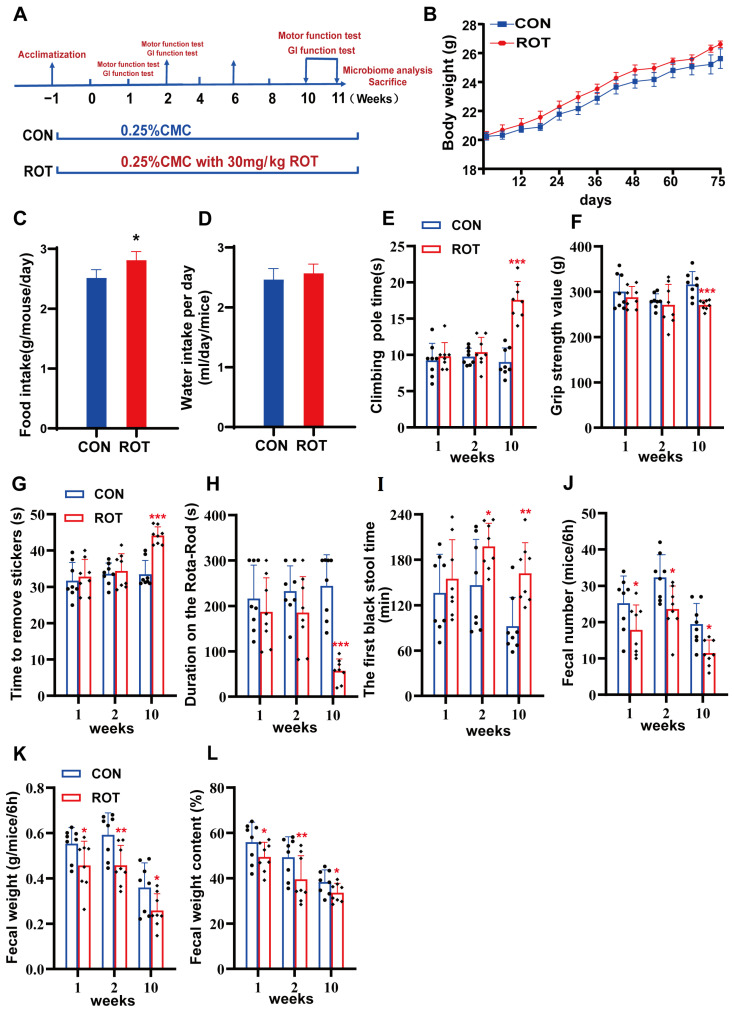
Effect of rotenone on motor and constipation symptoms in PD mice. (**A**) The flow chart of animal treatments. (**B**) The body weights of mice. (**C**) Food intake. (**D**) Water intake. (**E**) Pole test, PT. (**F**) Adhesive removal test, RST. (**G**) Grip strength test, GS. (**H**) Rota-Rod test, R-RT. (**I**) The first black stool time, FBST. (**J**) Fecal weight in 6 h, FW. (**K**) Fecal number in 6 h, FN. (**L**) Fecal water content, FWC. All data presented as mean ± SD, *n* = 10. * compared with the CON group. * *p* < 0.05, ** *p* < 0.01, and *** *p* <0.001.

Mice were treated with low-dose rotenone (30 mg/kg·BW, ROT) by gavage in this study (Figure 1A). During the induction of the established PD model, the body weight of both mice in the control group (CON) and mice in the ROT group increased slowly but without significant difference (Figure 1B, *p* > 0.05). Rotenonone administration resulted in a significant increase in food intake, while its effect on water intake was modest (Figure 1C,D, *p* < 0.05).

The present study evaluated the effects of rotenone on the locomotor ability of mice at three time points: weeks 1, 2, and 10. The results demonstrated that there was no statistically significant difference (*p* > 0.05) between the various behavioral tests of mice in the CON and ROT groups during the initial two weeks of rotenone treatment. The results of the test conducted in week 10 demonstrated significant motor deficits in mice treated with rotenone, as evidenced by prolonged times in both the pole-climbing and sticker–removal tasks (Figure 1E–G, *p* < 0.001). Additionally, there was a marked decrease in limb muscle strength (Figure 1F, *p* < 0.001) and a reduction in time spent on a rotating rod (Figure 1H, *p* < 0.05). These findings indicated that rotenone effectively induced motor symptoms of PD in mice.

In addition, rotenone successfully induced GI dysfunction in mice. The first black stool time (FBST) of mice in the rotenone group began to increase after 1 week of rotenone gavage, but there was no significant difference (Figure 1I, *p* > 0.05), whereas the fecal number (FN), fecal weight (FW), and fecal water content (FWC) were all significantly reduced as early as week 1 (Figure 1J–L, *p* < 0.05). After 2 weeks of rotenone treatment, mice in the ROT group showed a significant increase in FBST (Figure 1I, *p* < 0.05) and a significant decrease in FN, FW and FWC excreted within 6 h (Figure 1J–L, *p* < 0.05). These data suggest that rotenone-treated mice exhibited complete GI dysfunction as early as week 2, which preceded the detection of motor symptoms.

### 2.2. Effects of Rotenone on DA Neurons, α-Syn and 5-HT

**Figure 2:**
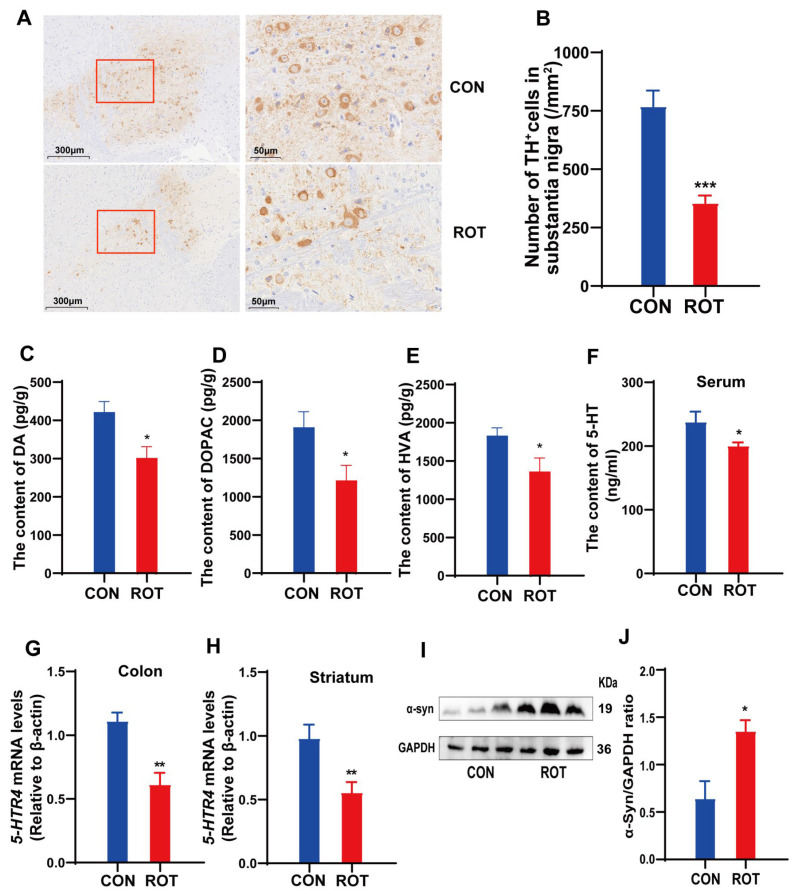
Effects of rotenone on DA neurons and related neurotransmitters in mice. (**A**) Representative pictures of TH^+^ neurons in SN. (**B**) Quantitative analysis of the optical density of TH immunohistostaining in SN. (**C**–**E**) The concentrations of DA, DOPAC and HVA in striatum. (**F**) The concentrations of 5-HT in serum. (**G**) 5-*HTR4* mRNA expression levels in striatum. (**H**) 5-*HTR4* mRNA expression levels in colon. (**I**) Representative western blot brands of α-syn in the midbrain containing the SN. (**J**) The density analysis results of α-syn in the midbrain containing the SN. All data presented as mean ± SEMs, *n* = 8 or *n* = 3 (Figure 2I). * compared with the CON group. * *p* < 0.05, ** *p* < 0.01, and *** *p* < 0.001.

Two histologic features of PD are DA neuron loss and a-syn accumulation. Tyrosine hydroxylase (TH) is the main enzyme for DA synthesis and is commonly used to label the number of DA neurons. At the same time, degenerative deficits in DA neurons also lead to decreased dopamine levels in the striatum. Immunohistochemical analysis demonstrated a significant reduction in the number of tyrosine hydroxylase-positive (TH^+^) cells in the substantia nigra (SN) of mice in the rotenone (ROT) group, showing an approximate 54% decrease compared to the CON group (Figure 2A,B, *p* < 0.001). Furthermore, the levels of dopamine (DA) and its metabolites, 3,4-dihydroxyphenylacetic acid (DOPAC) and homovanillic acid (HVA), in the striatum of the ROT group were significantly reduced by approximately 28.5% (Figure 2C, *p* < 0.05), 36.5% (Figure 2D, *p* < 0.05) and 25% (Figure 2E, *p* < 0.05), respectively, compared to the CON group. This indicated that rotenone caused the death of some DA neurons in the dense part of the substantia nigra (SN). As another pathological feature of PD in the SN, the level of α-syn protein expression was significantly increased in the SN of mice in the ROT group (Figure 2I,J, *p* < 0.05), which also suggested that low-dose rotenone was also successfully induced in the PD mouse model.

In addition, a correlation has been observed between decreased levels of 5-HT and the onset of PD [16]. Furthermore, 5-HT has been demonstrated to play a significant role in the regulation of defecation behavior and intestinal motility in animal models [17]. In this study, mice in the ROT group showed a significant reduction in serum levels of 5-HT (Figure 2F, *p* < 0.05) and mRNA expression levels of *5-HTR4* in the striatum and colon (Figure 2G,H, *p* < 0.01), suggesting that the 5-HT signaling system may be an important mediator of ROT-induced dyskinesia and constipation symptoms in PD.

### 2.3. Rotenone Affects Gene Expression of Aquaporins and GI Motility Factors in the Colon

**Figure 3:**
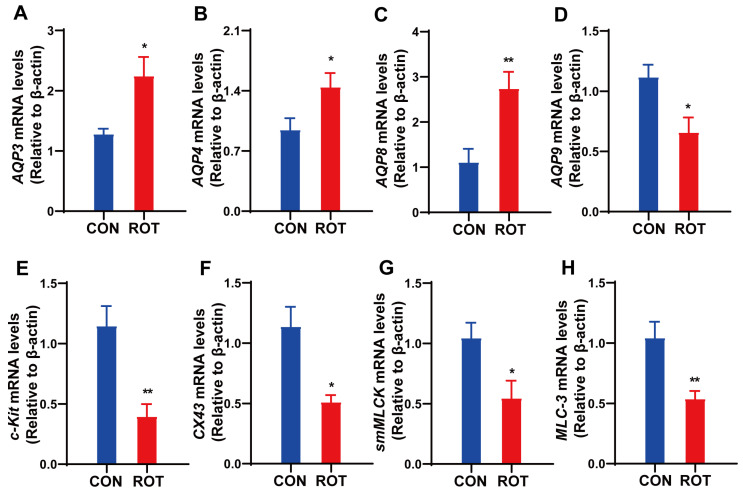
Effects of rotenone on the expression of aquaporins and GI motility factors in mouse colonic tissues. (**A**–**D**) The mRNA expression of *AQP3*, *AQP4*, *AQP8 and AQP9* in the colon, respectively. (**E**–**H**) The mRNA expression of stem cell factor receptor (*c-Kit*), Connexin 43 (*Cx43*), smooth muscle myosin light chain kinase (*smMLCK*) and myosin light chain 3 (*MLC-3*) in the colon. All data presented as mean ± SEMs, *n* = 10. * compared with the CON group. * *p* < 0.05, and ** *p* < 0.01.

Aquaporins (AQPs) play a critical role in the process of intestinal water absorption and metabolism. Abnormal expression of certain intestinal AQPs (AQP3, AQP4, AQP8 and AQP9) has been observed to have a significant impact on intestinal water metabolism, which may result in constipation [19]. Compared with the CON group, the mRNA expression levels of *AQP3*, *AQP4* and *AQP8* in the ROT group respectively increased by 75%, 53.19% and 148.18% (Figure 3A–C, *p* < 0.05), while the mRNA expression level of *AQP9* decreased by 40.54% (Figure 3D, *p* < 0.05). These results suggest that rotenone may regulate intestinal water metabolism and reduce fecal water content by modulating the gene expression levels of AQPs in mice.

Changes in GI motility are mainly regulated by smooth muscle contraction, and a decrease in its contractility also leads to a decrease in intestinal peristalsis, which in turn leads to constipation [20]. Smooth muscle myosin light chain kinase (smMLCK) is a key regulatory enzyme that controls the onset of smooth muscle contraction [21]. Stem cell factor receptor (c-Kit) is a specific marker for ICCs and plays an important role in improving the contractile and diastolic function of smooth muscle in the GI tract and regulating the GI dynamic system [22]. Connexin 43 (Cx43) is an important protein that forms gap junctions and is widely found between ICCs and SMCs and plays an important role in slow-wave transmission, and mutations and reductions in Cx43 lead to GI motility dysfunction [23]. In the present study, the mRNA expressions of *c-Kit*, *Cx43*, *smMLCK*,and myosin light chain 3 (*MLC-3*) in the colon of rotenone-induced PD mice were respectively reduced by 68.7%, 55.26%, 47.12% and 49.04 (Figure 3E–H, *p* < 0.05). These results suggest that rotenone may induce GI dyskinesia in mice by affecting the functions of ICCs and SMCs.

### 2.4. Rotenone Causes Inflammation and Barrier Damage in Both Mouse Colon and Brain Tissue

**Figure 4:**
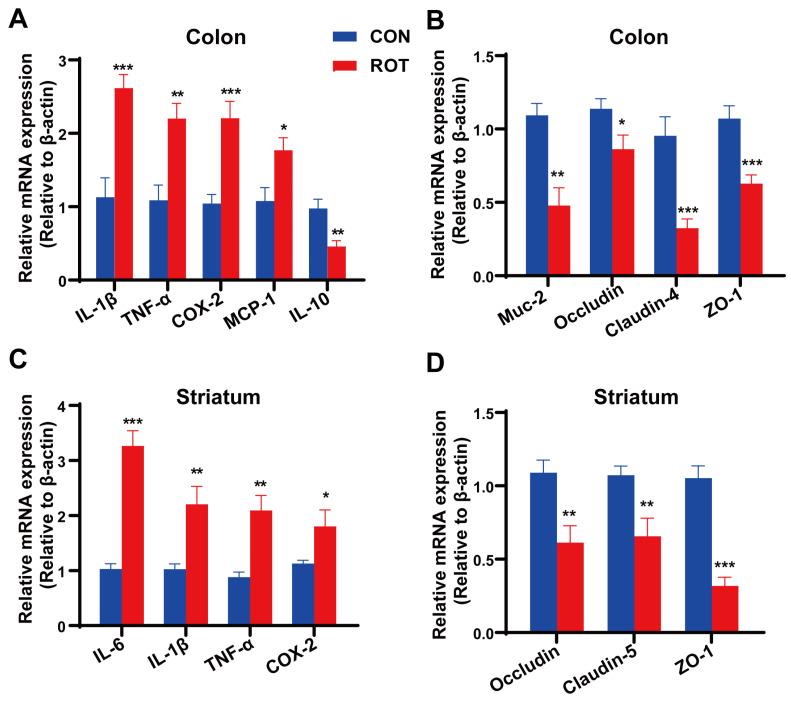
Effect of rotenone on mRNA expression of inflammatory cytokines and tight junction proteins in the colon and striatum. (**A**) The mRNA expression of *IL-1β*, *TNF-a*, *COX-2*, *MCP-1* and *IL-10* in the colon. (**B**) The mRNA expression of *Muc-2*, *Occludin*, *Claudin-4* and *ZO-1* in the colon. (**C**) The mRNA expression of neuroinflammatory markers (*IL-6*, *IL-1β*, *TNF-α* and *COX-2*) in Striatum. (**D**) The mRNA expression of *Occludin*, *Claudin-5* and *ZO-1* in striatum. All data presented as mean ± SEMs, *n* = 8. * compared with the CON group. * *p* < 0.05, ** *p* < 0.01, *** *p* < 0.001.

In colonic tissues, rotenone significantly increased the mRNA expression of pro-inflammatory cytokines *IL-1β*, *TNF-*α, *COX-2* and *MCP-1*, and significantly decreased the mRNA expression of anti-inflammatory cytokine IL-10 in mouse colonic tissues (Figure 4A, *p* < 0.01). In addition, the mRNA expression levels of intestinal barrier factors *Muc-2*, *Occludin*, *Claudin-4* and *ZO-1* were significantly reduced in the colon of mice in the ROT group (Figure 4B). These results suggest that rotenone induced motility and constipation symptoms along with inflammation in the colon of mice and contributed to impaired intestinal barrier function in these animals. In addition, in brain tissue (striatum), rotenone significantly increased the mRNA expression of pro-inflammatory factors, including *TNF-α*, *IL-6*, *IL-1β* and *COX-2* (Figure 4C, *p* < 0.01), while it simultaneously significantly decreased the mRNA expression of Occludin, *Claudin-5* and *ZO-1* (Figure 4D, *p* < 0.05). The results indicated that rotenone induced blood-brain barrier (BBB) damage and neuroinflammation.

### 2.5. Rotenone Inhibits the PI3K/AKT Pathway in SN and Colon

**Figure 5:**
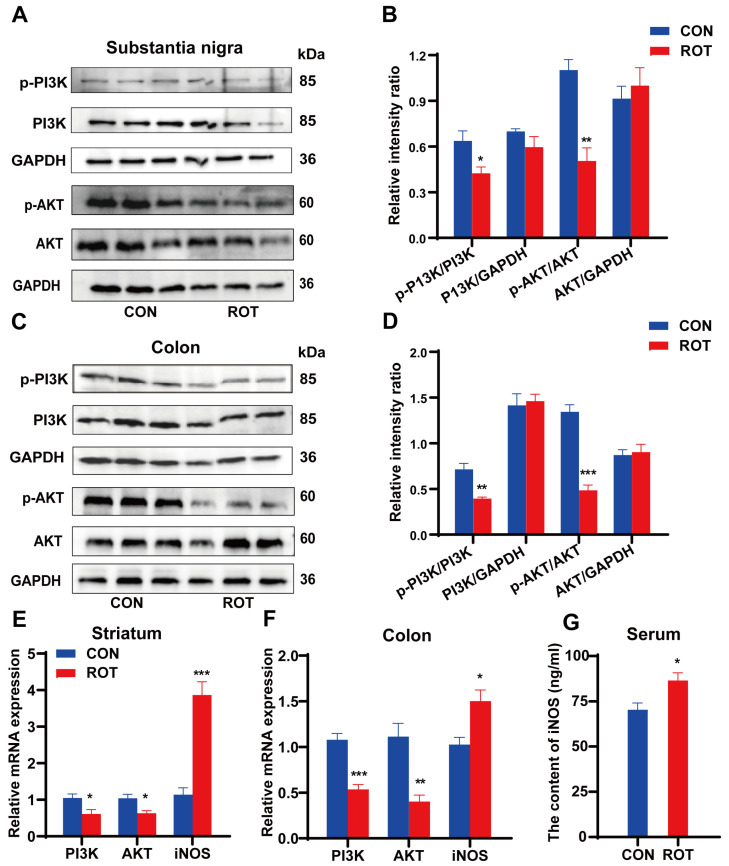
Inhibition effects of rotenone on the PI3K/AKT signaling pathway in the SN and colon. (**A**–**D**) Representative Western blot brands and the density analysis results of p-PI3K, PI3K, p-AKT and AKT in the SN and in the colon, respectively. (**E**,**F**) The mRNA expression levels of *PI3K*, *AKT* and *iNOS* in the striatum and colon, respectively. (**G**) The content of iNOS in serum. All data presented as mean ± SEMs, *n* = 3 or *n* = 8 (Figure 2E–G). * compared with the CON group. * *p* < 0.05, ** *p* < 0.01, *** *p* < 0.001.

The PI3K-AKT signaling pathway is one of the critical intracellular signaling pathways involved in cell proliferation, differentiation, metabolic regulation and cell survival. In the nervous system, this signaling pathway plays a pivotal role in neuronal survival, synaptic plasticity and neurotransmitter release [24]. A multitude of studies have demonstrated that p-AKT levels are markedly diminished in the SN of patients diagnosed with PD [25]. There is an escalating body of evidence suggesting that certain natural products possess the capacity to manifest neuroprotective effects by means of activating the PI3K-AKT pathway [26]. In this study, the protein expression levels of p-PI3K (*p* < 0.05) and p-AKT (*p* < 0.01) in the SN and colon of mice in the ROT group were significantly lower than those in the CON group (Figure 5A–D). Consistent with Western blot results, the mRNA expression levels of *PI3K* and *AKT* in the striatum and colon of ROT mice, respectively, decreased by 41.35%, 38.83%, 50.44% and 59.60% (Figure 5E,F, *p* < 0.05), suggesting that rotenone significantly inhibited the PI3K/AKT signaling pathway in both brain and intestinal tissues of mice.

Nitric oxide synthase (NOS), a downstream target of the PI3K/AKT signaling pathway, catalyzes the production of NO from L-arginine. Excess NO has been shown to cause intestinal smooth muscle relaxation, slowing GI peristalsis and prolonging colonic transit, which can lead to the development of constipation [10]. Our RT-qPCR and ELISA results showed that ROT stimulation increased the mRNA expression of *iNOS* in striatal and colonic tissues by 0.52-fold and 2.55-fold, respectively, and increased serum iNOS levels by 23% compared with CON (Figure 5E–G, *p* < 0.05). These results suggest that the PI3K/AKT/NOS signaling pathway may be the shared mechanism by which rotenone induces motor and constipation symptoms in PD.

### 2.6. Structural and Functional Characterization of Intestinal Microbiota in Rotenone-Induced PD Mice

**Figure 6:**
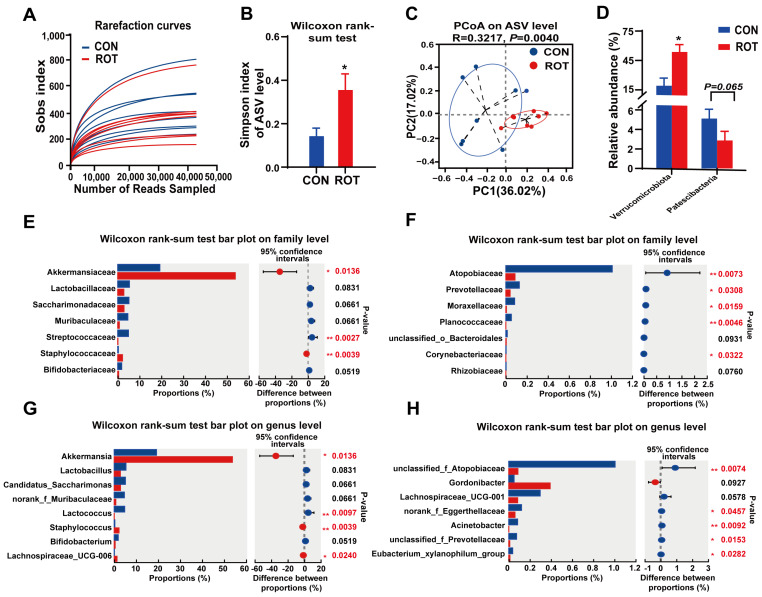
Effect of rotenone on the structure and composition of the mouse cecal microbial community. (**A**) The rarefaction curve of the Sobs index at the ASV level. (**B**) Simpson index at the ASV level. (**C**) PCoA analysis based on based on Bray–Curtis distance. (**D**–**H**) Relative abundances of different taxa at the phylum, family and genus levels, respectively, which were affected by the ROT. All data presented as mean ± SEMs, *n* = 8. * compared with the CON group. * *p* < 0.05, ** *p* < 0.01.

We proceeded to investigate the impact of rotenone on the composition of microbes in the cecum of mice by 16S rRNA gene sequencing. The Sobs sparse curve exhibited a flattening tendency as the number of sample sequencing reads attained 40,000, suggesting that the volume of data obtained from this sequencing was adequate (Figure 6A). Rotenone treatment significantly reduced the α-diversity of microbiota in the mouse cecum, including increased Simpson’s index (Figure 6B, *p* < 0.05) and decreased Shannon’s index (Figure S1, *p* = 0.066). Principal Coordinate Analysis (PCoA) results showed that samples from the CON and ROT groups were significantly separated (Figure 6C, *p* = 0.004).

Rotenone triggered substantial alterations in the microbial composition of mouse cecum contents. At the phylum level, rotenone treatment led to a marked increase in the relative abundance of *Verrucomicrobiota*, concurrent with a significant decrease in the relative abundance of *Patescibacteria* (Figure 6D, *p* < 0.05). At the family level, *Akkermansiaceae* and *Staphylococcaceae* exhibited a significant increase in relative abundance in ROT mice compared to CON mice (Figure 6E, *p* < 0.05). The relative abundance of *Streptococcaceae*, *Planococcaceae*, *Prevotellaceae*, *Atopobiaceae*, *Corynebacteriaceae*, *Moraxellaceae* and *Bifidobacteriaceae* decreased significantly (Figure 6E,F, *p* < 0.05). *Lactobacillaceae*, *Muribaculaceae*, and *Saccharimonadaceae* also exhibited a significant decrease in relative abundance in the rotenone group, though this decrease did not reach statistical significance (*p* > 0.05).

The differential microbial taxa in the CON and ROT groups at the genus level are shown in Figure 6G,H. The relative abundance of *Akkermansia*, *Staphylococcus* and *Lachnospiraceae*_UCG—006 was significantly increased in the ROT group of mice compared to the CON group (*p* < 0.05). Conversely, the relative abundances of *Lactobacillus*, unclassified_f_*Atopobiaceae*, *Acinetobacter*, norank_f_*Eggerthellaceae*, *Eubacterium_xylanophilum*_group, *Solibacillu* and unclassified_f_*Prevotellaceae* were significantly lower (*p* < 0.05). Furthermore, the relative abundance of *Bifidobacterium*, *Lachnospiraceae*_UCG—001, *Candidatus_Saccharimonas*, norank_f_*Muribaculaceae* and *Lactobacillus* were also significantly decreased in the ROT group; however, this decrease did not reach statistical significance (*p* > 0.05).

We further analyzed and predicted the effects of rotenone on the function of the mouse gut microbiota based on PICRUSt2. It was found that 13 KEGG level II pathways and 69 KEGG level III pathways were significantly altered by rotenone intervention (Figure S2). At KEGG level II, after rotenone administration, cell growth and death, nucleotide metabolism, exogenous biodegradation and metabolism, digestive system, immune disorders and biosynthesis of other secondary metabolites were significantly attenuated (*p* < 0.05). At the same time, glycan biosynthesis and metabolism, lipid metabolism and aging were significantly enhanced (*p* < 0.05). At KEGG level III, the ROT group differed significantly (*p* < 0.05) from the CON group in a variety of pathways, including galactose metabolism, lipopolysaccharide biosynthesis, sphingolipid biosynthesis, lysine degradation, sesquiterpene and triterpene biosynthesis, the PI3K-AKT signaling pathway and some pro-inflammatory pathways. In summary, rotenone treatment not only significantly affected the structure and composition of the intestinal microbial community in mice, but also had a significant effect on its function.

### 2.7. Correlations of Gut Microbial Taxa with Key Host Parameters in PD Mice

**Figure 7:**
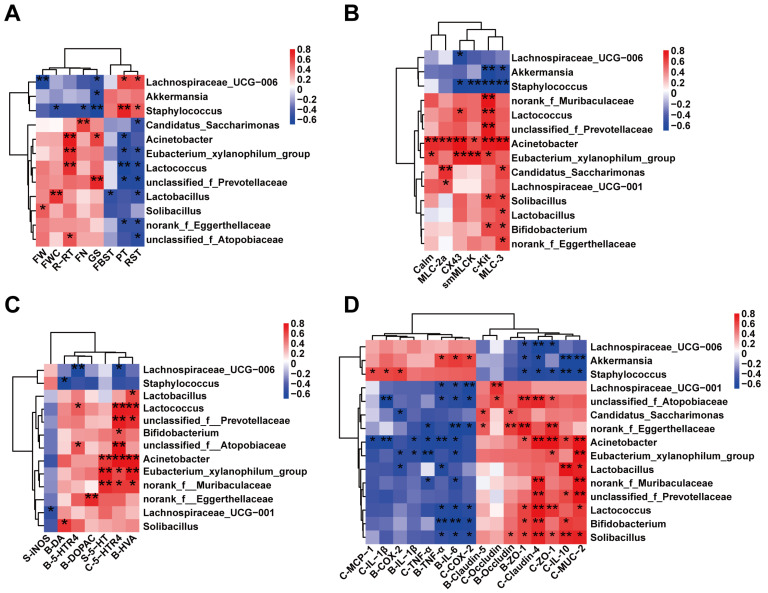
Heat maps showing correlations between specific gut bacteria and core host parameters. (**A**) Correlation analysis of differential microbes with motility phenotypes and defecation parameters in mice. (**B**) Correlation analysis of mouse differential microbes with GI motility factor and water channel protein gene expression. (**C**) Correlation analysis of mouse differential microbes with neurotransmitter levels. (**D**) Correlation analysis of mouse differential microbes with levels of inflammation and barrier-related genes. Bivariate correlations (*n* = 8), including correlations between gut bacteria and core host parameters. The color at each intersection indicates the value of the r coefficient; *p* values were adjusted for multiple testing according to Benjamini–Hochberg (BH) procedures were used to adjust *p* values for multiple testing; * indicates a significant correlation between these two parameters (* *p* < 0.05, ** *p* < 0.01). Abbreviations: (**A**) PT, Climbing pole time; GS, Grip strength value; R-RT, Duration on the Rota-Rod; RST, Time to remove stickers; FBST, the defecation time of the first black stool; FW, the fecal wet weight in 6 h; FN, the fecal number in 6 h; (**C**) S-iNOS, S-5-HT, the concentrations of iNOS, 5-HT in serum; B-5-HTR4, the mRNA expression levels of *5-HTR4* in the striatum; C-5-HTR4, the mRNA expression levels of *5-HTR4* in the colon; B-DA, B-HVA and B-DOPAC, the concentrations of DA, HVA and DOPAC in the striatum. (**D**) C-IL-1β, C-TNF-a, C-COX-2, C-MCP-1 and C-IL-10, the mRNA expression of *IL-1β*, *TNF-a*, *COX-2*, *MCP-1* and *IL-10* in the colon; C-Muc-2, C-Occludin, C-Claudin-4 and C-ZO-1, the mRNA expression of *Muc-2*, *Occludin*, *Claudin-4* and *ZO-1* in the colon; B-IL-6, B-IL-1β, B-TNF-α and B-COX-2, the mRNA expression of *IL-6*, *IL-1β*, *TNF-α* and *COX-2* in the striatum; B-Occludin, B-Claudin-5, and B-ZO-1, the mRNA expression of *Occludin*, *Claudin-5* and *ZO-1* in the striatum.

To reveal the important role of specific microbial taxa in the induction of PD symptoms by rotenone, we performed Spearman correlation analysis. As shown in Figure 7A, Staphylococcus and Lachnospiraceae_UCG—006 were positively correlated with both pole climbing (PT) and adhesive removal time (RST) and negatively correlated with grip strength (GS) in mice (*p* < 0.05); *Lactococcus*, unclassified_f_*Prevotellaceae*, *Eubacterium_xylanophilum*_group and norank_f_*Eggerthellaceae* were all negatively correlated with PT and RST, while *Lactococcus* and *Eubacterium_xylanophilum*_group were positively correlated with PT (*p* < 0.05). In addition, unclassified_f_*Atopobiaceae*, *Candidatus_Saccharimonas* and *Lactobacillus* were all negatively correlated with RST, and *Acinetobacter* was positively correlated with RST (*p* < 0.05). Among fecal parameters, *Staphylococcus* was negatively correlated with both fecal wet weight (FW) and fecal water content (FWC, *p* < 0.05); *Lactobacillus* was negatively correlated with the first black stool time (FBST) and positively correlated with FWC (*p* < 0.05); *Candidatus_Saccharimonas* had a strong positive correlation with fecal number (FN, *p* < 0.05); *Solibacillus* had a negative correlation with FW; and *Lachnospiraceae*_UCG—006 had a strong positive correlation with FW(*p* < 0.05). These results suggest that *Staphylococcus*, *Lachnospiraceae*_UCG—006 and *Lactobacillus* may play important roles in rotenone-induced motor dysfunction and constipation symptoms in mice.

As demonstrated in Figure 7B, the mRNA expression levels of genes associated with GI motility exhibited a predominantly positive correlation with the presence of *Acinetobacter*, *Eubacterium_xylanophilum*_group, *Solibacillus* and *Bifidobacterium*, and a predominantly negative correlation with *Staphylococcus* and *Akkermansia* (*p* < 0.05). This finding suggests the potential importance of *Acinetobacter*, *Eubacterium_xylanophilum*_group, *Staphylococcus*, *Solibacillus* and *Akkermansia* in the GI hyperdynamics induced by rotenone in mice.

As shown in Figure 7C, *Acinetobacter*, *Eubacterium_xylanophilum*_group and norank_f_*Muribaculaceae* were significantly and positively correlated with serum 5-HT levels, SN HVA levels and colonic *5-HTR4* mRNA expression (*p* < 0.05); meanwhile, *Lactococcus* and unclassified_f_*Prevotellaceae* were also significantly and positively correlated with nigral HVA levels and colonic *5-HTR4* expression (*p* < 0.05). In contrast, *Lachnospiraceae*_UCG—006 was negatively correlated with *5-HTR4* expression in both colonic tissue and striatum, and Lachnospiraceae_UCG—001 was negatively correlated with serum *iNOS* levels (*p* < 0.05). These results suggest that *Acinetobacter*, the *Eubacterium_xylanophilum*_group, norank_f_*Muribaculaceae*, *Lachnospiraceae*_UCG—006, *Lactococcus* and unclassified_f_*Prevotellaceae* are closely associated with abnormal neurotransmitter secretion induced by rotenone.

As shown in Figure 7D, the mRNA expression of barrier factors in brain tissue and intestine showed strong positive correlations with *Solibacillus*, *Acinetobacter*, *Lactococcus*, norank_f_*Eggerthellaceae*, unclassified_f_*Atopobiaceae*, unclassified_f_*Prevotellaceae* and *Bifidobacterium* (*p* < 0.05), whereas it was negatively correlated with *Staphylococcus*, *Akkermansia* and *Lachnospiraceae*_UCG—006 (*p* < 0.05). Expression of inflammation-related genes in brain tissue and intestine was strongly correlated with *Solibacillus*, *Lactococcus*, *Bifidobacterium*, unclassified_f_*Atopobiaceae*, *Lachnospiraceae*_UCG—001 and *Eubacterium_xylanophilum*_group were strongly negatively correlated and strongly positively correlated with *Akkermansia* and *Staphylococcus* (*p* < 0.05). These results suggest that *Solibacillus*, *Acinetobacter*, *Lactococcus*, *Bifidobacterium*, norank_f_*Eggerthellaceae*, unclassified_f_*Atopobiaceae*, *Akkermansia* and *Staphylococcus* play important roles in rotenone-induced brain and intestinal inflammation and barrier dysfunction in mice.

In summary, some specific taxa are closely related to locomotor ability, constipation symptoms, inflammation and barrier status of intestinal and brain tissues in mice. *Staphylococcus*, *Lachnospiraceae*_UCG—006 and *Akkermansia* may play a role in exacerbating dyskinesia and constipation symptoms during rotenone-induced PD, while *Acinetobacter*, *Solibacillus*, *Eubacterium_xylanophilum*_group, *Lactobacillus and Bifidobacterium* may be beneficial in maintaining exercise capacity, stimulating GI peristalsis, alleviating inflammation and barrier damage in mice.

## 3. Discussion

Rotenone has been widely used to induce PD in animal models. Rotenone treatment resulted in DA neuron neurodegeneration and accumulation of a-Syn in PD mice, leading to motor deficits [17], and also caused constipation and significantly delayed gastric emptying in SD rats [27]. In this study, we found a significant loss of TH^+^ cells in the SN and a marked increase in α- syn protein expression in the SN, indicating the successful establishment of the PD mouse model. Notably, low-dose rotenone-treated mice showed significant constipation symptoms in week 2, while dyskinesia was not significant. This suggests that oral rotenone-induced constipation symptoms precede motor symptoms, providing key evidence for the hypothesis that PD may originate in the gut.

Enteric neurons are crucial in the onset and progression of constipation. The accumulation of a-syn in the ENS correlates with a reduction in the number of neurons, potentially leading to dysfunction in intestinal fluid secretion and neurotransmitter activity, which further disrupts intestinal peristalsis and exacerbates constipation [28]. Neurotransmitters in the intestine are closely related to intestinal peristalsis. Serotonin (5-HT), predominantly secreted by enterochromaffin cells in the GI tract, interacts with the 5-HT4 receptor to elicit excitatory peristaltic signals [29]. 5-HT is expressed in both the central nervous system (CNS) and ENS. Studies have reported that the loss of 5-HT neurons in PD may precede the degeneration of dopaminergic neurons [30]. Clinical studies have demonstrated that plasma levels of 5-HT in PD patients are significantly lower, with 5-HT levels inversely correlated to non-motor symptom severity, including depression and constipation [31]. The 5-HT4 receptor has been shown to modulate dopamine release in the rat striatum and SN [32]. In our study, we found that rotenone treatment led to a marked decrease in serum 5-HT levels in mice, alongside a significant downregulation of *5-HT4R* expression in both mouse striatum and colon. These findings further underscore the pivotal role of 5-HT in the manifestation of motor and constipation symptoms associated with PD.

ICCs are a type of GI pacemaker cell, and their reduction and abnormal distribution may be an important factor in colonic motility dysfunction [33]. In the 6-OHDA-induced PD rat model, colonic Cx43 expression significantly decreased. Conversely, neurotrophic factor can regulate Cx43 activity to reduce reactive gliosis and inflammatory markers, promote colonic neuron survival and improve colonic motility in PD rats [34]. In the present study, we observed that rotenone gavage treatment significantly reduced the mRNA expression of *c-Kit*, *Cx43*, *smMLCK* and *MLC-3* in the colon of mice. This finding suggests that rotenone-induced constipation may be attributed to its impact on the function of ICCs and SMCs, leading to GI dyskinesia in mice.

The inflammatory environment has been demonstrated to enhance α-Syn aggregation [35]. Patients diagnosed with inflammatory bowel disease exhibit an elevated risk of developing PD [36]. Induced inflammatory mediators promote the infiltration of neutrophils and macrophages into the smooth muscle layer and enhance NO production. This reduces intestinal smooth muscle contraction, contributing to constipation [37]. Disruption of the BBB and intestinal barrier play a pivotal role in the progression of PD. GI tract inflammation due to microbiome dysbiosis can disrupt the intestinal barrier, increasing its permeability. Then, pro-inflammatory molecules in the circulation may further damage the BBB [38]. Previous studies have already found that rotenone may induce persistent intestinal inflammation and increase intestinal permeability, leading to impairment of the BBB and intestinal barrier [39]. In this study, rotenone caused significant changes in mouse brain and colon: decreased mRNA expression of tight junction proteins, increased mRNA expression of pro-inflammatory cytokines, and decreased mRNA expression of *IL-10*. These findings imply that rotenone-induced PD dyskinesia or constipation symptoms might not be merely a localized problem in the gut or brain group, but rather a manifestation of the overall dysfunction of the gut–brain axis.

The PI3K/AKT signaling pathway plays a pivotal role in regulating various cellular processes, including cell growth, differentiation, survival and apoptosis. AKT and p-AKT have been found to be significantly reduced in the SNpc of PD patients [40]. In a 6-OHDA-induced PD rat model, activation of the PI3K/AKT signaling pathway can ameliorate DA neuron damage in PD and alleviate oxidative stress and inflammation [41]. In this study, rotenone led to a substantial decrease in p-PI3K and p-AKT in the colon and SN, suggesting that the PI3K/AKT pathway was suppressed in PD mice. This finding is consistent with the previously observed blockage of PI3K/AKT pathway activation in PD animal models.

NOS, a downstream target of the PI3K/AKT signaling pathway, catalyzes NO production from L-arginine. Excessive NO induces intestinal smooth muscle relaxation, slows GI peristalsis, prolongs colonic transport and contributes to constipation [10]. The PI3K/AKT/NOS signaling pathway plays a pivotal role in the pathophysiology of slow transit constipation [42]. The Yiqi Jianpi Tongbian decoction can alleviate constipation symptoms in rats with slow transit constipation by modulating the PI3K/AKT/NOS signaling pathway [16]. In this study, we found that the mRNA expression of *iNOS* in colon tissue and the SN, as well as the content of iNOS in serum, increased significantly under the stimulation of rotenone. These findings suggest that the PI3K/AKT/NOS signaling pathway may be a shared mechanism of rotenone-induced PD motor symptoms and constipation symptoms.

5-HTR1, 5-HTR5, and other isoforms inhibit adenylate cyclase (AC) and cyclic adenosine phosphate (cAMP) production through Gi/o coupling, activating PI3K-AKT to regulate synaptic plasticity [43]. Furthermore, Tongbian decoction has been observed to alleviate constipation symptoms in rats by modulating 5-HT levels and enhancing the levels of p-PI3K/PI3K and p-AKT/AKT in colon tissue [44]. A stem cell factor (SCF) of c-kit ligand can interact with human c-Kit tyrosine residue 721 by inducing the PI3K regulatory subunit [45]. Pterostilbene ameliorated intestinal motility disorders in constipated mice by inhibiting apoptosis of ICCs through the PI3K/AKT pathway [11]. Activation of the PI3K/AKT signaling pathway and its downstream mTORC1 and mTORC2 complexes can regulate intracellular AQP3 levels [46]. Acupuncture combined with Liumo decoction can also reduce the expression of AQP3 and AQP8 in the colon by regulating the PI3K/AKT signaling pathway, achieving good laxative effect [47]. The PI3K/AKT pathway is crucial for the therapeutic effects of drugs on inflammation and barrier integrity. For example, Fasudil can reduce brain tissue inflammation-related factor expression by activating the PI3K/AKT pathway, protecting MPTP-induced PD model mice from DA neuron loss [48]. These findings suggest that rotenone affects 5-HT and its receptor, GI motility factors, aquaporins and intestinal inflammation/barrier-related factors, likely via the PI3K/AKT pathway, highlighting its role in rotenone-induced constipation in PD.

Dysregulation of gut microbiota has been observed in both patients with PD and PD-like animals [49]. Multiple fecal transplantation studies have also confirmed that changes in gut microbiota play an important role in the development of PD. The gut microbiota can exert an influence on the brain via the transmission of signals through the vagus nerve, thereby directly stimulating the electrical signals in the ENS and the dorsal vagus nerve, thus playing a neuroprotective role [50]. *Bifidobacterium animalis subsp. lactis* NJ241 can ameliorate behavioral impairments in murine models of PD by stimulating the production of short-chain fatty acids, which in turn promotes the secretion of glucagon-like peptide-1 [51]. In addition, the regulation of gut microbiota also plays an important role in the treatment of PD constipation symptoms. For example, some prebiotic combinations can significantly improve PD constipation symptoms [52].

Certain specific microbial taxa are pivotal in the pathogenesis of PD. In both PD individuals and rotenone-induced PD murine models, there was a marked increase in the relative abundance of *Akkermansia* [53]. Kleine Bardenhorst et al. proposed that elevated levels of *Akkermansia* in fecal samples could serve as a potential biomarker for PD identification [54]. Our investigation also revealed a significant rise in the relative abundance of *Akkermansia* within the cecal contents of rotenone-induced PD mice, exceeding 50%.

It has been suggested that an overabundance of it may deplete mucin, increasing intestinal permeability [55]. Decreased mucins mean a weakened intestinal barrier, exposing the body to bacterial endotoxins, which contributes to inflammation and increased α-Syn expression in PD [56]. In this study, we found that rotenone caused inflammation and barrier dysfunction in colon and brain tissue. The strong correlation of *Akkermansia* with tight junction proteins and inflammation-related genes in these tissues indicates that its overproliferation might be a crucial link in PD development.

*Lactobacillus* and *Bifidobacterium* represent the primary probiotic genera of interest. In patients with PD, there is a notable reduction in the abundance of *Lactobacillaceae* and *Bifidobacterium*, which have demonstrated neuroprotective properties among various animal models [57]. *Lactobacillus plantarum* CRL2130 has been found to prevent MPTP-induced dopaminergic neuronal cell death and mitigate motor deficits in PD mouse models [58]. *Bifidobacterium animalis subsp. lactis* Probio-M8 (M8) intake improves GI symptoms and motor symptoms associated with changes in the host gut microbiome in PD mice [59]. Additionally, *Bifidobacterium animalis subsp. animalis* IM386 and *Lactiplantibacillus* plantarum WCFS1 have been shown to influence actin cytoskeletal dynamics and tight junction integrity by enhancing protein kinase C and PI3K/AKT anti-apoptotic signaling pathways, thereby facilitating the restoration of the intestinal epithelial barrier [60]. It is widely known that *Lactobacillus* and *Bifidobacterium* help mitigate constipation. In this study, rotenone treatment significantly reduced their relative abundance. Correlation analyses further indicate that these probiotics may play a role in preserving exercise capacity, promoting GI motility, and alleviating inflammation and barrier dysfunction in murine models.

In patients with PD, there is a significant increase in *Staphylococcus* abundance [61]. Likewise, a significant increase in *Staphylococcus* in MPTP-induced PD model mice [62]. The administration of sea cucumber fucosylated kondroitin sulfate may alleviate inflammation associated with dysbiosis by reducing *Staphylococcus* levels, thereby mitigating PD symptoms [63]. Furthermore, studies have reported an upsurge in *Lachnospiraceae* abundance in mice exposed to rotenone, with elevated *Lachnospiraceae* levels also observed in PD patients presenting with constipation [63,64]. Importantly, in this investigation, the ROT group mice demonstrated a substantial increase in the relative abundance of both *Staphylococcus* and *Lachnospiraceae_UCG*—*006*. Correlation analyses further suggest that increased *Staphylococcus* and *Lachnospiraceae_UCG*—*006* abundance may exacerbate GI motility disorders, heighten CNS and GI inflammation and impair barrier integrity, indicating their pivotal role in PD symptom development in rotenone-treated mice.

It is noteworthy that rotenone significantly influenced the relative abundances of several low-abundance microbial taxa, including *Acinetobacter*, *Eubacterium_xylanophilum*_group and *Solibacillus*. Correlation analyses revealed a strong association between these taxa and rotenone-induced motor deficits, symptoms of constipation, GI motility alterations, and abnormal neurotransmitter secretion, as well as inflammation and barrier dysfunction. A notable reduction in *Acinetobacter* abundance was observed in patients with functional constipation in Japan [65]. Similarly, patients with chronic constipation exhibiting small intestinal bacterial overgrowth also demonstrated a significant decrease in *Acinetobacter* levels. Furthermore, flaxseed treatment has been shown to enhance bowel function in elderly patients with chronic constipation, and concurrently modify the intestinal microecological landscape and increase the relative abundance of *Acinetobacter* [66]. *Eubacterium* is a significant intestinal bacterium that has been identified in the colon of healthy humans. Notably, Korean red ginseng significantly increased the abundance of *Eubacterium* that can produce anti-inflammatory substances while improving the MPTP-induced PD symptoms [67]. *Lycium ruthenicum* Murray have been demonstrated to increase the relative abundance of the *norank_f_Eubacterium_coprostanoligenes*_group, contributing to their neuroprotective effects [68]. This implies that Acinetobacter and the *Eubacterium_xylanophilum*_group may significantly contribute to rotenone-induced PD pathogenesis. In summary, the gut microbiota may serve as a shared pathway through which rotenone triggers motor deficits and constipation linked to PD. Specific microbial taxa could have unique contributions to this mechanism.

## 4. Materials and Methods

### 4.1. Animals and Experimental Design

Six-week-old male C57BL/6 mice (*n* = 20), weighing 18–20 g, were procured from Beijing Vital River Laboratory Animal Technology Co., Ltd., Beijing, China. The mice were housed in an environment with a temperature of 23 ± 2 °C, relative humidity of 40–60%, and a 12 h light/dark cycle. All mice were acclimatized for a period of seven days prior to the commencement of the experiment. During the test period, mice were provided with ad libitum access to food and water. Prior to the initiation of the behavioral test, the mice underwent a two-day training regimen encompassing the following tests: the pole test, the rotarod test, the grip strength test and the adhesive removal test.

Rotenone (Sigma-Aldrich, St. Louis, MI, USA) was dissolved in sodium carboxymethylcellulose (CMC-Na, Sigma-Aldrich, St. Louis, MI, USA) containing 0.5% carboxymethylcellulose. The sequence of animal experiments is illustrated in Figure 1A. Twenty mice were randomly divided into two groups: a control group (CON, *n* = 10) and a model group (ROT, *n* = 10). The model group was gavaged daily with rotenone (30 mg/kg·BW), while the control mice were given an equal volume of 0.5% CMC-Na. Behavioral tests and defecation tests were performed at weeks 1, 2 and 10 after the experiments were conducted, and all mice were subsequently sacrificed for further analysis.

### 4.2. Behavioral Test

In this study, four primary behavioral assessments were conducted to evaluate motor function in mice. Each test was repeated thrice, with an interval of one hour between each iteration.

#### 4.2.1. Pole Test

The operational methodology of the pole test is delineated in [32], which is herein outlined in summary. The climbing pole (SA111, SANS, Nanjing, China) is 50 cm in height, with a diameter of 1 cm. It is topped with a ball with a diameter of 35 mm. The climbing pole was positioned at an angle of 45°, and during the test, the mouse was placed on the ball. The time required for the mouse to climb to the bottom was meticulously recorded. Each mouse underwent three trials, and the mean value was documented. An interval of 1 h was maintained between each trial.

#### 4.2.2. Adhesive Removal Test

Each mouse was assigned to a clean cage and permitted to move freely for one minute to acclimatize. Prior to the formal experiment, a circular adhesive label (0.3 cm × 0.4 cm) was gently affixed to the forepaw of each mouse. The time required for complete removal of the label was subsequently recorded for each mouse. The experimenter performed three consecutive trials on each mouse, allowing 30 min between each trial [39].

#### 4.2.3. Grip Strength Test

The mice were positioned on the shelf of the Grip Strength Meter (SA415, SANS, Nanjing, China). Once their limbs firmly grasped the shelf, the mice were pulled by their tails with uniform force in a horizontal direction until they released the shelf. The maximum grip force of the mice was recorded during this process. Each mouse was measured on three separate occasions, and the mean value was calculated. Three independent tests were performed at 1 h intervals for each mouse [32].

#### 4.2.4. Rotarod Test

At the commencement of the formal experiment, the rotational velocity of the bar was set at 35 r/min, and the duration of the test was set to 180 s. Prior to each trial, the mice were acclimated to the rotating rod (SA120M, SANS, Nanjing, China) for 30 s before the experiment commenced. The time from the initiation of the rotating rod to its cessation within 180 s was documented, and this period was designated as the locomotor latency of the mice. Each mouse was subjected to three trials, with an interval of one hour between each trial [39].

### 4.3. Defecation Test

Prior to conducting the defecation test, it is imperative that all groups of mice be subjected to food withdrawal but not water deprivation. Following a 12 h fast (8: 30 a.m.), each group of mice was gavaged according to standard protocol. Following a 30 min period, each mouse was gavaged with ink, and the mouse was individually placed in a cage. The first black stool time (FBST) was recorded for each mouse, and the number of feces (FN) and the fecal weight (FW) excreted by each mouse during the 6 h period were recorded. Immediately following the observation period, fecal samples were collected and dried at 80 °C until constant weight. The fecal water content (FWC) was subsequently calculated [23].

### 4.4. Immunohistochemical Staining

Paraffin-embedded sections were dewaxed and rehydrated in xylene. Following this procedure, the sections were repaired in EDTA repair solution. Then, the sections were treated with 3% H_2_O_2_ to remove endogenous peroxidase activity from the tissue. Non-specific antibody binding was blocked by incubating the sections with bovine serum albumin V. This was followed by an overnight incubation with anti-TH (Abcam, ab137869, Cambridge, UK) antibody at 4 °C. Subsequently, the sections were incubated with biotin-conjugated secondary antibody (1:200, GB23303, Servicebio, Wuhan, China) at 37 °C for 30 min. The sections were initially stained with DAB (Boster, Wuhan, China) and subsequently re-stained with hematoxylin. Thereafter, the sections were dehydrated and sealed. The number of positive cells was analyzed by using Image Pro Plus 6.0 software. Each section was evaluated from six randomly selected areas.

### 4.5. Enzyme-Linked Immunosorbent Assay (ELISA)

After the mice were euthanized, blood samples were collected, incubated at 37 °C for 30 min and centrifuged at 3500 rpm for 10 min at 4 °C. The serum was aliquoted and stored at −80 °C. For striatum tissue, approximately 100 mg of tissue was weighed and rinsed, minced, homogenized, freeze-thawed twice and centrifuged, and the supernatant was stored at −80 °C. ELISA kits were utilized to ascertain the levels of dopamine (DA) (Wuhan Huamei Biological Engineering Co., Ltd., Wuhan, China) and 3,4-dihydroxyphenylacetic acid (DOPAC) in the striatum of mice, and homovanillic acid (HVA) (Nanjing Camilo Bioengineering Co., Ltd., Nanjing, China) and the serum levels of 5-hydroxytryptamine (5-HT) and iNOS of mice (Wuhan Huamei Biological Engineering Co., Ltd., Wuhan, China). All experimental procedures were performed according to the manufacturer’s recommended instructions.

### 4.6. Western Blotting

Tissues were lysed using RIPA lysis buffer (Applygen, Beijing, China) containing a mixture of protease and phosphatase inhibitors. The lysates were then subjected to centrifugation at 12,000× *g* for 20 min at 4 °C to extract total protein. The protein concentration was detected using the BCA protein assay kit (Beyotime, Shanghai, China). The protein blotting procedure was executed by means of the sodium dodecyl sulfate polyacrylamide gel electrophoresis method, as previously delineated in our prior publication [40]. The membranes were subsequently subjected to an overnight incubation at 4 °C with the following primary antibodies: anti-a-syn antibody (1:500, A22598, Proteintech, Wuhan, China), anti-PI3K p85 antibody (1:1000, ab191606, Abcam, Cambridge, UK), p-PI3 Kinase p85 (Tyr458)/p55 (Tyr199) antibody (1:1000, #4228, CST, Boston, MA, USA), anti-AKT antibody (1:5000, 80816-1-RR, Proteintech, Wuhan, China), anti-AKT1 (pS473) + AKT2 (pS47 4) + AKT3 (pS472) (1:1000, ab192623, Abcam, Cambridge, UK). The membranes were then incubated with the corresponding secondary antibodies, such as HRP anti-goat anti-rabbit antibody (1:2500, AS031, Abclonal, Wuhan, China), for 1 h at room temperature. The blots were then subjected to visualization using the Novex™ ECL Chemiluminescent Substrate Kit (WP20005, Thermo Fisher Scientific, Waltham, MA, USA). The densities of the blots were subsequently analyzed and counted using Image J 1.5.4 software.

### 4.7. RNA Extraction, Reverse Transcription and Real-Time Quantitative PCR

Total RNA was isolated from the colon tissues using the TaKaRa MiniBEST Universal RNA Extraction Kit (9767, Takara, Kyoto, Japan) following the manufacturer’s instructions. Subsequently, RNA reverse transcription was performed with the PrimeScript™ RT reagent Kit with gDNA Eraser (Perfect Real Time, RR047A, Takara, Kyoto, Japan), and the quantitative PCR analysis of gene expression using TB Green^®^ Premix Ex TaqTM II (Tli RNaseH Plus, RR820A, Takara, Kyoto, Japan). The relative amount of the target mRNA was normalized to the β-actin level, and the results were calculated using the 2^−∆∆Ct^ method. The primer sequences can be found in Table S1.

### 4.8. DNA Extraction and 16S rRNA Gene Sequencing of Cecal Contents

Subsequent to the euthanization of the mice in week 10, the cecum contents were collected for freezing and stored at −80 °C for subsequent analysis. All cecum samples were subjected to microbial community total genomic DNA extraction in accordance with the instructions of the E.Z.N.A.^®^ soil DNA kit (Omega Bio-tek, Norcross, GA, USA). The quality of the extracted genomic DNA was subsequently checked by agarose gel electrophoresis with 1% agarose, and the concentration and purity of DNA were determined using the NanoDrop2000 (Thermo Fisher Scientific, Waltham, MA, USA). The extracted DNA was then utilized as a template for the PCR amplification. The PCR amplification was carried out using the upstream primer, 338F (5′-ACTCCTACGGGGAGGCAGCAG-3′), and the downstream primer, 806R (5′-GGACTACHVGGGTWTCTAAT-3′), both of which carry barcode sequences. The PCR conditions and the purification of the PCR products were performed using a previously described method [23]. Purified amplicons were pooled in equimolar amounts and paired-end sequenced on an Illumina PE300 platform (Illumina, San Diego, CA, USA) according to the standard protocols by Majorbio Bio-Pharm Technology Co., Ltd. (Shanghai, China).

### 4.9. Bioinformatics Analysis

Bioinformatic analysis of the gut microbiota was carried out using the Majorbio Cloud platform (https://cloud.majorbio.com, accessed on 8 October 2024). Based on the ASV information, rarefaction curves and alpha diversity indices, including observed ASVs and the Simpson index, were calculated with Mothur vl.30.1. The similarity among the microbial communities in different samples was determined by principal coordinate analysis (PCoA) based on Bray–Curtis dissimilarity using the Vegan v2.5-3 package.

### 4.10. Data Analysis

The analysis of the data was conducted using GraphPad Prism 9.0 software, and the results were expressed as the mean ± standard errors (SEMs). The two groups of data were compared using an unpaired two-tailed *t*-test, and the results were considered statistically significant at the time of the *p* < 0.05. Multivariate analysis was performed using the cloud platform of Shanghai Majorbio Biomedical Technology Co. Ltd., Shanghai, China (https://www.majorbio.com, accessed on 8 October 2024); bivariate correlations between host parameters and gut microbiota were analyzed using SPSS 25.0 software and the Spearman method; and heat maps were constructed using the Lianchuan Biological Cloud Platform (https://www.omicstudio.cn/home, accessed on 5 December 2024).

## 5. Conclusions

**Figure 8:**
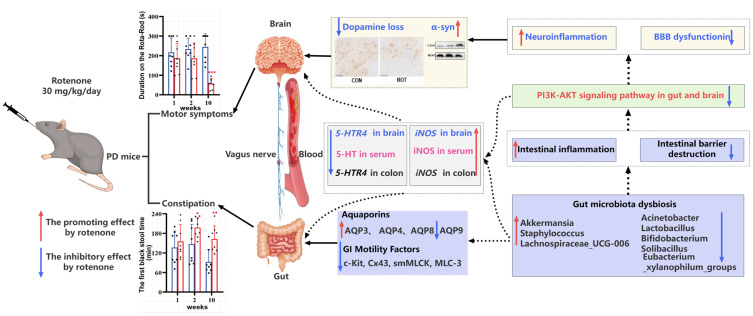
The mechanisms of rotenone-induced parkinsonism with constipation.

The present study used low-dose rotenone gavage to create a mouse model of PD and found that constipation symptoms appeared earlier than dyskinesia in mice. Low-dose rotenone gavage for 10 weeks not only caused loss of DA neurons and accumulation of α-syn, but also significantly suppressed the expression of 5-HT, *5-HT4R* and GI motility factors in colon. Both colon and brain showed severe inflammation and barrier dysfunction induced by rotenone; the PI3K/AKT pathway in the SN and colon was also significantly inhibited. The structure, composition and function of the gut microbial community in the mouse cecum were also significantly altered by rotenone. Some specific taxa of microbes were strongly associated with motility, constipation symptoms, inflammation and the barrier status of gut and brain in PD mice. *Akkermansia*, *Staphylococcus* and *Lachnospiraceae_UCG*—*006* may play a role in exacerbating constipation symptoms, whereas *Acinetobacter*, *Lactobacillus, Bifidobacterium*, *Solibacillus* and *Eubacterium_xylanophilum*_group may be beneficial in maintaining GI peristalsis and body motor function and alleviating inflammation and barrier damage. This suggests that rotenone may cause parkinsonism with constipation symptoms in mice by disrupting the gut micro-ecosystem and inhibiting the PI3K-AKT pathway and GI motility (Figure 8). These findings provide direction for further elucidation of the mechanism by which rotenone induces the production of constipation symptoms. In the future, we plan to lower the dose of rotenone and study the motor and constipation symptoms of PD at different time points, so as to explore the hypothesis of the intestinal origin of PD.

## Data Availability

The raw reads of the 16S rRNA gene sequence data were deposited into the NCBI Sequence Read Archive (SRA) database under BioProject accession number PRJNA1212295.

## Supplementary Materials

The following supporting information can be downloaded at: https://www.mdpi.com/article/10.3390/ijms26052079/s1.

## Abbreviations

The following abbreviations are used in this manuscript:

PD	Parkinson’s disease
FBST	The first black stool time
FN	Fecal number
FW	Fecal wet weight
FWC	Fecal water content
GI	Gastrointestinal
Muc-2	Mucin 2
ZO-1	Zonula occludens protein 1
IL-1β	Interleukin-1β
IL-6	Interleukin-6
PT	Climbing pole time
GS	Grip strength value
R-RT	Duration on the Rota-Rod
RST	Time to remove stickers
SN	Substantia nigra
TH	Tyrosine hydroxylase
TNF-α	Tumour necrosis factor-α
MCP-1	Monocyte chemotactic protein-1
COX-2	Cyclooxygenase-2
DA	Dopamine
DOPAC	3,4-Dihydroxyphenylacetic acid
HVA	Homovanillic acid
PI3K	Phosphatidylinositol 3-kinase
AKT	Protein Kinase B
5-HT	5-hydroxytryptamine
BBB	Blood–brain barrier
ICCs	Interstitial cells of Cajal
NOS	Nitric oxide synthase
iNOS	Induced nitric oxide synthases
c-Kit	Stem cell factor receptor
SMCs	Smooth muscle cells
smMLCK	Smooth muscle myosin light chain kinase
Cx43	Connexin 43
MLC-3	Myosin light chain 3
