# Supplementary material for: Rotenone Induces Parkinsonism with Constipation Symptoms in Mice by Disrupting the Gut Microecosystem, Inhibiting the PI3K-AKT Signaling Pathway and Gastrointestinal Motility
Source: Int J Mol Sci. 2025 Feb 27;26(5):2079. doi: 10.3390/ijms26052079 (PMC11899888; doi:10.3390/ijms26052079)
Supplement: Supplementary file 1 [file ijms-26-02079-s001.zip › ijms-3471054-supplementary.pdf]

Supplementary Material

Rotenone Induces Parkinsonism with Constipation Symptoms in Mice by Disrupting the Gut Microecosystem, Inhibiting the PI3K-AKT Signaling Pathway and Gastrointestinal Motility

Li Liu [1,2,3,†], Yan Zhao [4,†], Weixing Yang [2], Yuqin Fan [2], Lixiang Han [2], Jun Sheng [1], Yang Tian [1] and Xiaoyu Gao [1,2,3,*]

[1] Yunnan Key Laboratory of Precision Nutrition and Personalized Food Manufacturing, Yunnan Agricultural University, Kunming 650201, China; 2022210085@stu.ynau.edu.cn (L.L.); shengj@ynau.edu.cn (J.S.); tianyang@ynau.edu.cn (Y.T.)
[2] College of Food Science and Technology, Yunnan Agricultural University, Kunming 650201, China; 2021110006@stu.ynau.edu.cn(W.Y.); 15348746213@163.com (Y.F.); 15368025745@163.com (L.H.)
[3] Engineering Research Center of Development and Utilization of Food and Drug Homologous Resources, Ministry of Education, Yunnan Agricultural University, Kunming 650201, China
[4] Division of Science and Technology, Yunnan Agricultural University, Kunming 650201, China; 2021013@ynau.edu.cn
* Correspondence: 2018014@ynau.edu.cn
† These authors contributed equally to this work.

Table S1. Paired primers for qPCR.

Name	Primer sequence	
	Forward	Reverse
Muc-2	ATGCCCACCTCCTCAAAGAC	GTAGTTTCCGTTGGAACAGTGAA
ZO-1	GCCGCTAAGAGCACAGCAA	TCCCCACTCTGAAAATGAGGA
Occludin	ATGTCCGGCCGATGCTCTC	TTTGGCTGCTCTTGGGTCTGTAT
Claudin 4	GCAGAGCACAGGTCAGATGCA	AGGGCAGGTCCTGGAGAATGT
5HT4R	AGTTCCAACGAGGGTTTCAGG	CAGCAGGTTGCCCAAGATG
VIP	AGTGTGCTGTTCTCTCAGTCG	GCCATTTTCTGCTAAGGGATTCT
COX-2	AACATTCCTTCCCCCAGCAA	TCTATCACTGGCATCCGCTG
IL-1β	TCCATGAGCTTTGTACAAGGA	AGCCCATACTTTAGGAAGACA
TNF-α	AGACCCTCACACTCAGATCA	TCTTTGAGATCCATGCCGTTG
IL-10	GCTCTTACTGACTGGCATGAG	CGCAGCTCTAGGAGCATGTG
iNOS	GACATTACGACCCCTCCCAC	GACATTACGACCCCTCCCAC
IL-18	GACTCTTGCGTCAACTTCAAGG	CAGGCTGTCTTTTGTCAACGA
MCP-1	TTAAAAACCTGGATCGGAACCAA	GCATTAGCTTCAGATTTACGGGT
Caspase-1	ACAAGGCACGGGACCTATG	TCCCAGTCAGTCCTGGAAATG
Caspase-3	GACTGGAAAGCCGAAACTC	GGCAAGCCATCTCCTCATC
NLRP3	TTCGGAGATTGTGGTTGGG	GTCACCGAGGGCGTTGTC
AQP3	GCTTTTGGCTTCGCTGTCAC	TAGATGGGCAGCTTGATCCAG
AQP4	CTTTCTGGAAGGCAGTCTCAG	CCACACCGAGCAAAACAAAGAT
AQP8	ACCACTGGAACTTCCACTG	AATGAAGCACCTAATGAGCAG
AQP9	TTGGCATTTACTATGACGGACTC	CCCCTCGTATTGCAGACAAAA
CX43	GCAAGGGTGAGGAGGGGTA	CCTCTGAAGGCATTTCATAAGCC
MLCK	GGACTTTCAGCCTTGTGAT	CGCAAAACTTCCTTCTACTGTC
MLC-2a	GGCACAACGTGGCTCTTCTAA	TGCAGATGATCCCATCCCTGT
MLC3	ATGGGTGCTGAAATCCGTCAT	CCGCCACTAGCATCTCTACTTC
Calm	GATGGCACCATTACCACCAAG	CGCTGTCTGTATCCTTCATCTTT
smMLCK	CCATATCCGAGAAATGCTGGG	TGGGATTCCAGGTATGTATCACC
c-Kit	CAGAAACCCATGTATGAAGT	CTTTCCAAAACTCAGCCTGT
Akt	ACGTGGTGAATACATCAAGACC	GCTACAGAGAAATTGTTCAGGGG
Pi3k	GGTCACTTCTTGTTCTGGTTCC	ATGGTTTCGTTGGATAGGACTG
β-actin	CGGACACGGACAGGATTGACA	CCAGACAAATCGCTCCACCAACT

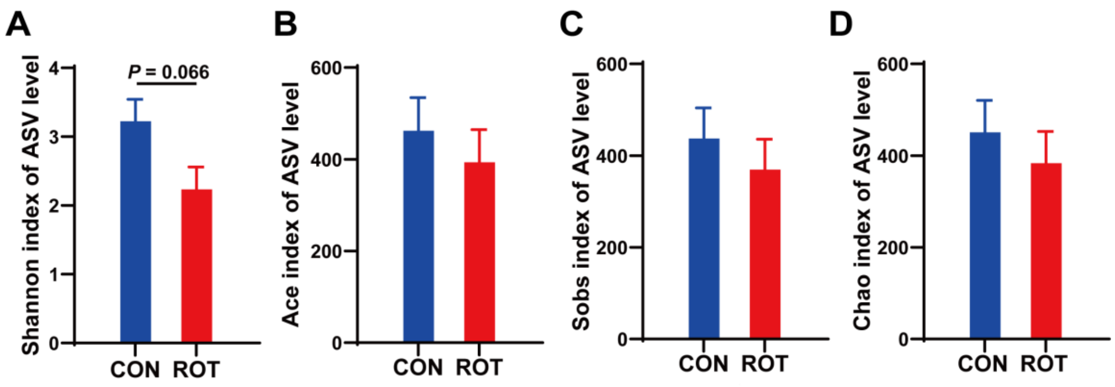

Figure S1. Effect of rotenone on on Alpha diversity of the gut microbiota in mice.

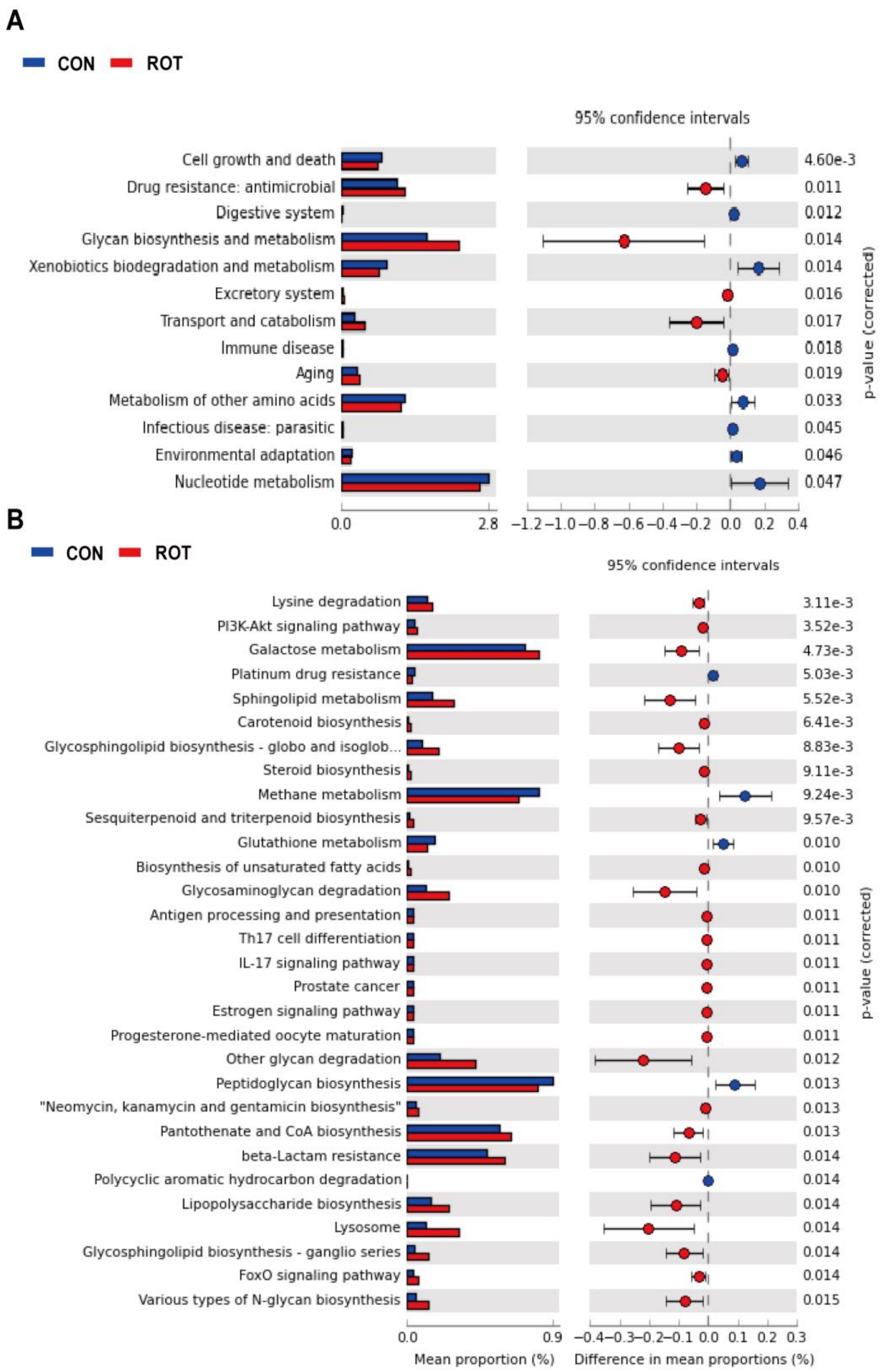

Figure S2. KEGG pathway function prediction analysis based on PICRUSt2. (**A**) KEGG pathway in level II (*P-value* correct <0.05, FDR) , (**B**) KEGG pathway in level III (TOP30 of *P-value* correct <0.05, FDR).
